# The burden of laboratory-confirmed influenza infection in Lebanon between 2008 and 2016: a single tertiary care center experience

**DOI:** 10.1186/s12879-020-05013-7

**Published:** 2020-05-12

**Authors:** Aia Assaf-Casals, Zeina Saleh, Sarah Khafaja, Danielle Fayad, Hady Ezzeddine, Mohammad Saleh, Sarah Chamseddine, Rouba Sayegh, Sima L. Sharara, Ahmad Chmaisse, Souha S. Kanj, Zeina Kanafani, Rima Hanna-Wakim, George F. Araj, Rami Mahfouz, Reiko Saito, Hiroshi Suzuki, Hassan Zaraket, Ghassan S. Dbaibo

**Affiliations:** 1 Center for Infectious Diseases Research, Faculty of Medicine, American University of Beirut, PO Box: 11-0236, Riad El-Solh, Beirut, 1107 2020 Lebanon; 2 Division of Pediatric Infectious Diseases, Department of Pediatrics and Adolescent Medicine, Faculty of Medicine, American University of Beirut Medical Center, PO Box: 11-0236, Riad El-Solh, Beirut, 1107 2020 Lebanon; 3 Division of Infectious Diseases, Department of Internal Medicine, Faculty of Medicine, American University of Beirut, PO Box: 11-0236, Riad El-Solh, Beirut, 1107 2020 Lebanon; 4 Department of Pathology and Laboratory Medicine, Faculty of Medicine, American University of Beirut, PO Box: 11-0236, Riad El-Solh, Beirut, 1107 2020 Lebanon; 5 Department of Public Health at Niigata University, Niigata, Japan; 6 Department of Experimental Pathology, Immunology & Microbiology, Faculty of Medicine, American University of Beirut, PO Box: 11-0236, Riad El-Solh, Beirut, 1107 2020 Lebanon

**Keywords:** Influenza, Hospitalization, Morbidity, Mortality, Antiviral use

## Abstract

**Background:**

Influenza is a major cause of morbidity and mortality worldwide. Following the 2009 pandemic, there was widened interest in studying influenza burden in all regions. However, since data from the World Health Organization (WHO) Middle East and North Africa (MENA) region remain limited, we aimed to contribute to the understanding of influenza burden in Lebanon.

**Methods:**

A retrospective chart review extending over a period of 8 seasons from Jan 1st, 2008 till June 30th, 2016 at a tertiary care center in Beirut was performed. All cases confirmed to have influenza based on rapid antigen detection or/and polymerase chain reaction on a respiratory sample were included for analysis. Data on epidemiology, clinical presentation, complications, antiviral use and mortality were collected for analysis.

**Results:**

A total of 1829 cases of laboratory-confirmed influenza were identified. Average annual positivity rate was 14% (positive tests over total requested). Both influenza A and B co-circulated in each season with predominance of influenza A. Influenza virus started circulating in December and peaked in January and February. The age group of 19–50 years accounted for the largest proportion of cases (22.5%) followed by the age group of 5–19 years (18%). Pneumonia was the most common complication reported in 33% of cases. Mortality reached 3.8%. The two extremes of age (< 2 years and ≥ 65 years) were associated with a more severe course of disease, hospitalization, intensive care unit (ICU) admission, complications, and mortality rate. Of all the identified cases, 26% were hospitalized. Moderate-to-severe disease was more likely in influenza B cases but no difference in mortality was reported between the two types. Antivirals were prescribed in 68.8% and antibiotics in 41% of cases. There seemed to be an increasing trend in the number of diagnosed and hospitalized cases over the years of the study.

**Conclusion:**

Patients with laboratory-confirmed influenza at our center had a high rate of hospitalization and mortality. A population based prospective surveillance study is needed to better estimate the burden of Influenza in Lebanon that would help formulate a policy on influenza control.

## Background

Acute respiratory infections constitute a major cause of morbidity and mortality globally [1]. According to the updated 2004 World Health Organization (WHO) report, the death toll from acute lower respiratory infections caused by bacterial and viral infections reached 4.2 million and accounted for 7.2% of all deaths worldwide [1, 2]. Data from the Global Burden of Disease study estimated that the mortality rate from lower respiratory tract infections due to influenza reached 0.9 per 100,000 population in the WHO Middle East and North Africa (MENA) region and the hospitalization rate ranged from 100 to 299 per 100,000 population [3].

Influenza is a major cause of both influenza-like illness (ILI) and more importantly severe acute respiratory infection (SARI) across the globe [4, 5]. Several studies in different regions of the world have indicated that influenza is a significant underlying cause for hospitalization of patients with acute respiratory infection (ARI) [6–9].

Infection with influenza may manifest in a wide spectrum of illness levels ranging from asymptomatic infection to death. Different studies reported asymptomatic cases to account for 14% up to 77% of influenza infections [10–12]. The major cause for hospital admission due to influenza is the development of pneumonia, which could be either primary viral or secondary to bacterial co-infection, and acute respiratory distress syndrome [13, 14]. In severe cases of influenza infection, death may ensue even in the absence of comorbidities [15].

Annual seasonal influenza vaccination can reduce the chances of getting infected and subsequently prevent spread of the virus. Vaccines are available in two forms: the inactivated influenza vaccine (IIV), which is administered intramuscularly or intradermally, and the live attenuated influenza vaccine, administered intranasally, (LAIV). Vaccination is recommended by the Centers for Disease Control, USA, annually to everyone above 6 months of age and should be given before influenza season commences, usually during early fall or late summer [16]. However, in many countries, especially developing ones, there is a lack of data on the epidemiology of influenza disease leading to a lack of appreciation of its true burden by public health authorities and impeding the introduction of influenza vaccine on national vaccination schedules. Accordingly, the WHO recommended the generation of country-specific epidemiological data in order to identify high priority groups, emphasizing the inclusion of pregnant women, and the optimal timing of vaccination against influenza [17].

Given the epidemic nature of the illness, there has been an increasing interest in the burden resulting from its outbreaks in addition to concerns about its association with myocardial infarctions, glycemic variation in diabetic patients, and the exacerbation of other chronic illnesses [1, 18–27]. While data on the epidemiology and severity of this disease has been primarily obtained from studies in temperate developed countries, developing regions including the MENA region have also begun investigating this burden in their hospitals allowing the formation of more complete global data [28].

In Lebanon, the influenza burden of disease is unknown. Since 2008, surveillance of influenza virus among ILI patients at the American University of Beirut Medical Center (AUBMC), was performed at the Center for Infectious Diseases Research (CIDR) [29–33]. The Ministry of Public Health started reporting influenza activity as part of its SARI surveillance since 2015. Whereas these surveillance programs furnish important information about influenza activity in Lebanon, they do not reflect the total burden of disease of influenza. In fact, an ideal study to look at the burden of influenza diseases would be population-based, prospective, and armed with a highly sensitive and specific test for influenza to be conducted both in outpatient and inpatient settings. However, such a study is not feasible and too expensive to conduct. An alternative approach that we chose was to conduct a retrospective review on laboratory-proven influenza at our center, a major academic medical center in Lebanon, where both inpatient and outpatient services exist. The objective of this review is to contribute to the understanding of the burden of disease of influenza in Lebanon. This may help inform public policy about prevention of influenza infections.

## Methods

### Study design

This is a retrospective review of patients who tested positive for influenza virus at AUBMC, a tertiary care medical center located in Beirut, Lebanon, between January 1, 2008 and June 30, 2016. Samples were collected from these patients and tested for influenza A and B viruses by rapid diagnostic test and/or by real-time reverse-transcriptase polymerase chain reaction (RT-PCR). Information already collected for patients diagnosed to have influenza A or B covering all the seasons from 2008 till 2016 who were previously enrolled in three ongoing surveillance studies at the CIDR at AUBMC (Surveillance of respiratory virus infections among pediatric cancer patients at the children’s cancer center of Lebanon [34], Viral infections in patients at the children’s cancer center of Lebanon and Epidemiologic surveillance of respiratory viral infections in Lebanon [29–35] were also included. Influenza season in Lebanon starts in late November and lasts through the month of April.

#### Biological specimens

1. Samples were either taken by nasopharyngeal swab or nasal wash and tested directly (at bed side - as part of the ILI surveillance studies) using one of the rapid diagnostics test kits or were sent in sterile normal saline to AUBMC laboratory and tested within 24 h. Deep tracheal aspirates were mostly used for testing patients who were intubated.
2. Storage: For those samples obtained from patients participating in the IRB-approved surveillance studies since 2008, a second sample was taken (after obtaining consent) and was transported in virus transport media, stored at 4 °C for no more than 24 h or at -20 °C if taken over the weekend. These samples were ultimately stored at − 80 °C until their transfer to the Department of Public Health at Niigata University, Japan for multiplex polymerase chain reaction and viral culture.

#### Laboratory analysis

1. Different rapid antigen detection kits were used across the different seasons. These included: Rapid FLU virus test kit Capilia™ Tauns, Japan; Quick-Ex flu A/B rapid influenza test Denka Seiken, Japan; Quick-Ex flu A/B & RSV rapid influenza test Denka Seiken, Japan; and BD Directigen™ EZ Flu A + B (Becton Dickinson, Cockeysville, Md.). Testing was performed following the manufacturer’s instructions.
2. Polymerase chain reaction: real-time reverse-transcriptase (RT) polymerase chain reaction (PCR) using the GENEXPERT (Cepheid) platform was used at AUBMC Clinical Molecular Biology Laboratory [33].
3. Only 2 patients were diagnosed by serology testing (Influenza A IgG titers cutoff < 1:10, our patients had a titer level of 1:5120). This was during the 2009 influenza pandemic.

### Identification of patients

All patients were identified retrospectively through medical records at AUBMC and through the parallel ILI surveillance studies for influenza conducted during the study period. These surveillance studies enrolled patients with symptoms fitting the ILI case definition, presenting as outpatients or inpatients, and tested them for influenza using rapid diagnosis test (RDT) and then RT-PCR. The list of remaining patients was retrieved by the medical coder by looking at the following lab codes: “8108” for rapid influenza A & B antigen detection and “8988” for Flu PCR. This was done through the Department of Pathology and Laboratory Medicine by sending a Statistical Information Request. Data from each chart was collected and recorded on a case report form (CRF). Medical records with the discharge diagnosis of influenza were also retrieved.

The CRF included information on demographic data (gender, age, residence, etc.), social history, medical history, influenza vaccination history, clinical symptoms, hospital course, severity parameters (O_2_ requirement, intensive care admission, mechanical ventilation, complications), radiology findings, targeted laboratory results, samples dates, method of influenza detection, use of antibiotics/antivirals, hospital stay and outcomes.

### Inclusion criteria

1. Any patient regardless of age or hospital admission status who was tested for influenza using either nasal wash/swab followed by PCR or RDT, or by serology at AUBMC lab or who was enrolled in the surveillance studies conducted at CIDR during the time period extending from January 1, 2008 till June 30, 2016 was included in the initial subject count. To note that surveillance studies were interrupted during the 2012/2013 and 2015/2016 seasons. Each influenza season included cases starting July till the end of June of the following year.
2. Patients who tested positive for influenza A, B or both were included for detailed review and analysis in the current study.

Patients were categorized into 7 age groups: below 2 years, [2–5 years[, [5–10 years[, [10–19 years[, [19–50 years[, [50–65 years[and above 65 years.

### Exclusion criteria

1. Lack of testing for influenza

### Definition

Moderate to severe influenza disease was defined as (1) fever > 39 degree Celsius (39 °C), and/or (2) physician-verified shortness of breath, pulmonary congestion, pneumonia, bronchiolitis, bronchitis, wheezing, croup, or acute otitis media, and/or (3) physician-diagnosed serious extra-pulmonary complication of influenza, including myositis, encephalitis, seizure, and/or myocarditis [36].

### Statistical analysis

The Statistical Package for Social Sciences (SPSS) program, version 22.0 for Windows was used for data analysis (IBM, Armonk, NY). Simple descriptive statistics was used to describe patients’ demographics, morbidity and mortality. Bivariate and multivariate analyses of risk factors for mortality in cases of laboratory proven influenza in hospitalized patients was analyzed by Pearson’s Chi-square test or Fisher’s exact test (when the number of patients in a subgroup was less than 5). Continuous risk factors were analyzed with student *t* test. Statistical significance was considered below a type-1 error threshold (alpha level) of 0.05. Following that a multivariate logistic regression model comprised of all risk factors with unadjusted *p*-value < 0.2 (neurological disorders, malignancy, moderate to severe disease, radiologically confirmed pneumonia, proven bacterial co-infection and proven viral co-infection) was constructed and reported.

## Results

During the study period spanning 8 influenza seasons from January 1st, 2008 till June 30th, 2016, a total of 11,288 patients presented to AUBMC with a respiratory illness for which they were tested for influenza infection. Out of all patients tested, 1829 patients (16%) had a positive test result for influenza; 542 influenza cases (29.6%) were identified during the 2009 influenza pandemic. Of the 1829 positive cases, 1529 (84%) were caused by influenza A, 242 cases (13%) by influenza B, and 55 cases (3%) had co-infection with both types (type was not reported in three cases). During the 2009 pandemic, 97.6% of the infections were caused by influenza A and 2% were co-infected. However, subtyping was done for only 35 cases during the pandemic, of which 28 cases were caused by influenza A (H1N1)pdm09.

### Demographic and clinical characteristics of the patients

Of the 1829 cases, 916 (50.1%) cases were female and 72 cases were pregnant; 24% of female patients in the childbearing age (Table 1). Most of the patients resided in Beirut and Mount Lebanon governorates (73%) reflecting the population served by our center. Patients were divided into 7 age groups: the highest proportion (22.5%) was for adults between 19 and 50 years old, while only 9.5% were below 2 years old. At least one underlying comorbidity was present in 45.8% of cases reviewed and 31% had two or more comorbidities. The most common underlying chronic disease among adults was cardiovascular diseases (33%), malignancy (15%) and diabetes mellitus (14.8%). However, in the younger age groups (< 19 years) the most common underlying chronic diseases were asthma (16%) and malignancy (10.4%). Data on the immunization status against influenza was available for 456 cases; 104 of these patients (22.8%) had received influenza vaccine in the past 12 months at the time of diagnosis with influenza infection.

**Table 1:** Demographic and clinical characteristics of laboratory-confirmed influenza patients

	Number (%)
**Gender (*n*** **= 1829)**	
*Male*	913 (49.9)
*Female*	916 (50.1)
**Age groups (*n*= 1829**)	
*[0–2 years*	173 (9.5)
*[2–5 years*	240 (13.1)
*[5–10 years*	338 (18.5)
*[10–19 years*	328 (17.9)
*[19–50 years*	411 (22.5)
*[50–65 years*	164 (9.0)
*≥ 65 years*	175 (9.6)
**Presence of underlying comorbidities (*n*** **= 1303)**	597 (45.8)
**Number of underlying comorbidities (*n*= 1303)**	
*None*	706 (54.2)
*One*	412 (31.6)
*Multiple comorbidities*	185 (14.2)
**Underlying comorbidities (*n*= 1303)**	
*Cardiovascular disease*	222 (17.0)
*Chronic pulmonary disease*	54 (4.1)
*Asthma*	160 (12.3)
*Immunosuppressive state (other than malignancy)*	19 (1.5)
*Diabetes Mellitus*	96 (7.4)
*Renal disease*	52 (4.0)
*Neuromuscular disorders*	12 (0.9)
*Neurological disorders*	58 (4.5)
*Malignancy*	164 (12.6)
*Hemoglobinopathy*	30 (2.3)
**Time interval between symptom onset and speicmen collection (*n*** **= 1368)**	
*0–2 days*	882 (64.5)
*3–4 days*	338 (24.7)
*> 4 days*	148 (10.8)
**Influenza Vaccine (*n*** **= 456)**	104 (22.8)
**Pregnant among childbearing age females (*N*** **= 322)**^a^	72 (22.4)

^a^ This denominator represents the number of females in childbearing age for whom data on pregnancy was available

The most common presenting symptoms were respiratory (96%) with cough constituting 91.5% of these symptoms. Fever was present in 89% of cases; 78% of febrile patients with data on highest temperature recorded had high-grade fever ≥39 °C. Gastrointestinal symptoms were present in 33.7% including vomiting (47%), diarrhea (32%) and abdominal pain (29.6%). Myalgia was reported in 31% of patients. Other less common symptoms (13.6%) included conjunctivitis, otitis, neurological events, confusion and dizziness (Table S1).

Complications secondary to influenza infection were captured when available. Almost 24% (295/1245) of patients developed at least one complication. Pneumonia, confirmed by the presence of infiltrates or consolidation on radiological examination, was the most common complication (241/295;81.6%). Chest X-ray was done in 233 of these cases and detected the presence of pneumonia in 218 cases (90.4%); the remaining 15 patients (6.6%) had a clear chest X-ray but had abnormal findings on computed tomography (CT). In eight patients, only a CT was done initially and detected the presence of pneumonia. However, the treating physician did not request an imaging study in 1095 subjects (60%). When imaging was requested, pneumonia was detected in 32.6% of cases (241/739). The highest rate of pneumonia cases was in the older age groups: ≥65 years old (27.4%) and 50–65 years (19.1%) (*P-value* < 0.001, using Pearson’s Chi-square test). Other reported complications were croup (3 cases), shock (20 cases) of which 20% were below 2 years and 25% were above 65 years of age, sepsis (23 cases) of which 39% were ≥ 65 years, myocardial infarction (4 cases), renal failure (12 cases, all adults), acute respiratory distress syndrome (16 cases, 25% were < 2 years and 37.5% were between 19 and 50 years old), encephalopathy (6 cases), hemorrhagic pneumonia (1 case), stroke (2 cases), seizures (7 cases) and liver failure (5 cases; all adults).

Moderate to severe influenza was encountered in 591 cases (42%) with a significant *P*-value (< 0.001) between different age groups. Almost half of the patients below 5 years (50.5%) and above 50 years (51.6%) had moderate to severe disease while around a third (28%) of those 10 to 50 years did (Table 2). When comparing percentages of moderate to severe disease within each type of influenza detected, influenza B infection was associated with a slightly higher incidence (49.8%) of moderate to severe disease compared to influenza A infection (40.9%) (*P*-value *=* 0.054).

**Table 2:** Severity parameters of laboratory-confirmed influenza cases according to different age groups

	Total n (%)	[0–2[ n (%)	[2–5[ n (%)	[5–10[ n (%)	[10–19[ n (%)	[19–50[ n (%)	[50–65[ n (%)	≥65 n (%)	*p*-value
**Moderate to severe disease (*N*** **= 1399)**	591 (42.2)	80 (51.9)	103 (52.0)	95 (45.5)	60 (28.0)	91 (29.3)	73 (49.7)	89 (53.6)	**< 0.001**
**Hospitalization rate (*N*** **= 1787)**	475 (26.6)	57 (34.5)	37 (16.2)	24 (7.3)	24 (7.5)	103 (25.5)	99 (60.4)	131 (74.9)	**< 0.001**
**ICU admission (*N*** **= 1767)**	70 (4.0)	13 (8.0)	6 (2.6)	1 (0.3)	2 (0.6)	14 (3.5)	11 (6.7)	23 (13.2)	**< 0.001**
**Oxygen supplementation (*N*** **= 1613)**	121 (7.5)	13 (9.4)	8 (4.1)	6 (2.1)	3 (1.1)	17 (4.4)	25 (15.5)	49 (28.3)	**< 0.001**
**Mechanical ventilation (N = 1767)**	35 (2.0)	8 (4.9)	5 (2.2)	1 (0.3)	2 (0.6)	8 (2.0)	4 (2.4)	7 (4.0)	**0.002**^a^
**Complication rate (*N*** **= 1245)**	295 (23.7)	45 (34.4)	36 (22.1)	20 (11.6)	16 (9.6)	51 (16.9)	52 (35.6)	75 (45.5)	**< 0.001**
**Mortality rate in hospitalized patients (*N*** **= 469)**	18 (3.8)	2 (3.6)	2 (5.7)	0 (0.0)	0 (0.0)	3 (2.9)	5 (5.1)	6 (4.6)	0.909^a^
**Duration of ICU stay in days, mean (**±SD) **(*N*** **= 70)**	9.2 (±8.9)	8.2 (±**5.2)**	8.7 (8.8)	22^b^	5.5 (±6.4)	12.6 (±12.3)	7.7 (±6.6)	8.2 (9.3)	0.533
**Duration of hospital stay in days, mean (**±SD) **(*N*** **= 473)**^c^	7.0 (±8.8)	8.5 (±10.3)	8.1 (±8.7)	7.2 (13.1)	6.6 (±5.0)	6.5 (±8.7)	6.4 (±6.8)	7.0 (±8.8)	0.788

Pearson’s Chi-Square test was used (no expected count less than 5)

^a^Fisher’s exact test was used when expected count was less than 5

^b^Only one patient was admitted to ICU in this age group

^c^Two patients were excluded; one stayed in 113 days and the other stayed for 262 days related to their underlying medical condition (Neuromuscular disease and cerebral palsy)

To note that a different denominator was used to calculate the rate of each variable. The denominator depended on the number of cases with available data on the studied variable

### Influenza type A, B and co-infection

Influenza detection was done using RDT or RT-PCR except for 2 cases where diagnosis was made by serology because the clinical picture was highly suggestive of influenza infection despite negative RDT and PCR. These 2 cases had an Influenza A IgG dilution titers of 1:5120 (negative < 1;10). Most of the cases were diagnosed by RDT (1546 samples, 84.5%) while 144 cases (7.9%) were detected by PCR. In 137 cases (7.5%), both RDT and PCR were used at the same time for detection. In these cases, PCR was likely requested for subtyping. Subtyping was done for 245 cases only, of which 138 cases were H1N1pdm09, 7 cases were H1N1 Brisbane, 61 cases were H3N2 and 3 cases were co-infected with H1N1pdm09 and H3N2.As for influenza B cases, 20 were Victoria lineage, 14 were Yamagata and 2 were Shanghai lineage.

Characteristics of patients with laboratory-confirmed influenza by virus type are illustrated in (Table 3). There was a significant variation in the incidence of each type of influenza virus through the different influenza seasons and among different age groups. Mean hospital stay duration was significantly higher in those who were co-infected with both types of influenza (17 days vs 7 days). Co-infection with both A and B viruses was significantly associated with higher incidence of ICU admission. Antibiotic and antiviral prescription was similar for both types and for co-infection. Death was equally reported for both types of influenza viruses (*P*-value = 1).

**Table 3:** Characteristics of laboratory confirmed influenza cases by virus type

**Characteristics**	**Influenza A,*N*** **= 1529**	**Influenza B,*N*** **= 242**	**Co-infection A&B,*N*** **= 55**	***p*-value**
	**n (%)**	**n (%)**	**n (%)**	
**Season (*N*** **= 1826)**				**< 0.001**^b^
*Jan 2008-Jun 2008*	12 (66.7)	6 (33.3)	0 (0.0)	
*2008–2009*	47 (78.3)	6 (10.0)	7 (11.7)	
*2009–2010*	529 (97.6)	2 (0.4)	11 (2.0)	
*2010–2011*	43 (58.9)	25 (34.2)	5 (6.8)	
*2011–2012*	92 (84.4)	2 (1.8)	15 (13.8)	
*2012–2013*	93 (73.8)	27 (21.4)	6 (4.8)	
*2013–2014*	183 (89.3)	17 (8.3)	5 (2.4)	
*2014–2015*	153 (64.3)	82 (34.5)	3 (1.3)	
*2015–2016*	377 (82.9)	75 (16.5)	3 (0.7)	
**Age group (*N*= 1826)**				
*[0–2 years [*	134 (8.8)	28 (11.6)	11 (20.0)	**0.039**^b^
*[2–5 years [*	205 (13.4)	25 (10.3)	9 (16.4)	
*[5–10 years [*	289 (18.9)	44 (18.2)	5 (9.1)	
*[10–19 years [*	283 (18.5)	37 (15.3)	7 (12.7)	
*[19–50 years [*	340 (22.2)	56 (23.1)	14 (25.5)	
*[50–65 years [*	138 (9.0)	25 (10.3)	1 (1.8)	
*≥ 65 years*	140 (9.2)	27 (11.2)	8 (14.5)	
**Gender (*N*= 1826)**				0.992
*Male*	764 (50.0)	121 (50.0)	27 (49.1)	
*Female*	765 (50.0)	121 (50.0)	28 (50.9)	
**Underlying medical conditions (*N*** **= 1300)**				
*Any*	511 (47.8)	89 (45.2)	15 (44.1)	0.74
*Cardiovascular disease*	179 (16.7)	35 (17.8)	8 (23.5)	0.563
*Chronic pulmonary disease*	51 (4.8)	3 (1.5)	0 (0.0)	0.054
*Asthma*	135 (12.6)	20 (10.2)	5 (14.7)	0.543
*Immunosuppressive state (other than malignancy)*	16 (1.5)	3 (1.5)	0 (0.0)	1.000^a^
*Diabetes Mellitus*	81 (7.6)	13 (6.6)	2 (5.9)	0.938^a^
*Renal disease*	43 (4.0)	8 (4.1)	1 (2.9)	1.000^a^
*Neuromuscular disorders*	8 (0.7)	4 (2.0)	0 (0.0)	0.204^a^
*Neurological disorders*	47 (4.4)	8 (4.1)	3 (8.8)	0.395^a^
*Malignancy*	136 (12.7)	27 (13.7)	1 (2.9)	0.213^a^
*Hemoglobinopathy*	27 (2.5)	3 (1.5)	0 (0.0)	0.749^a^
**Antibiotic prescription (*N*** **= 1201)**	395 (40.0)	87 (47.8)	12 (37.5)	0.134
**Antiviral prescription (*N*** **= 1191)**	684 (69.8)	119 (65.7)	17 (56.7)	0.192
**Radiologically confirmed pneumonia (*N*** **= 739)**	189 (31.5)	45 (39.1)	7 (29.2)	0.260
**Hospitalization rate (*N*** **= 1784)**	381 (25.4)	84 (36.2)	10 (18.5)	**0.001**
**Characteristics**	**Influenza A,*N*** **= 381**	**Influenza B,*N*** **= 84**	**Co-infection A&B,*N*** **= 10**	***p*-value**
	**n (%)**	**n (%)**	**n (%)**	
**Progression of illness and in-hospital outcome**				
*Moderate to severe disease (N = 470)*	224 (59.4)	59 (71.1)	7 (70.0)	0.129^a^
*ICU admission (N = 475)*	51 (13.4)	15 (17.9)	4 (40.0)	**0.042**^a^
*Oxygen therapy (N = 470)*	87 (23.0)	20 (24.4)	4 (40.0)	0.399^a^
*Mortality (N = 470)*	15 (4.0)	3 (3.7)	0 (0.0)	1.000^a^
**Hospital stay duration, mean (±SD) (N = 473)**^c^				
	6.8 (±8.4)	6.8 (±6.7)	16.7 (±23.8)	**0.02**

Percentages are expressed within each influenza type, except for the season variable in which percentages are expressed within each season.

*Pearson’s Chi-Square test was used (no expected count less than 5)*.

*Independent Sample-t test is used to compare means*.

^a^ Fisher’s exact test was used when expected count was less than 5

^b^ Monte Carlo estimate was used when the data set was too large to compute exact significance

^c^ Two patients were excluded; one stayed in 113 days and the other stayed for 262 days related to their underlying medical condition (Neuromuscular disease and cerebral palsy)

**Fig. 1:**
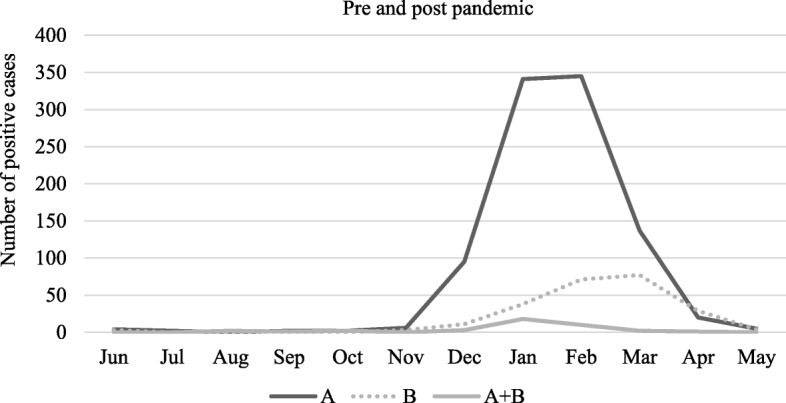
Influenza A and B virus circulation by month

Figure 1 shows the monthly distribution of influenza A and B through the various seasons. Influenza A was mostly detected during the months of January (37.4%) and February (34.8%). Another peak, however, is seen in November (51.2%) mostly during the 2009 pandemic. Influenza B tended to peak later during the month of March (32.1%). Over the different seasons, influenza infections started by the month of November and peaked by January except during the 2009 pandemic when a first wave was seen during the summer months and peaked in August and then another significant wave that started in October and peaked in November.

### Progression of illness and in-hospital outcome of laboratory-confirmed influenza patients

A total of 475 cases (26.6%, *N* = 1787) required hospital admission for management. Table 2 shows a comparison of the course of hospital stay among the different age groups with laboratory-confirmed influenza. The highest hospitalization rates were among the elderly ≥65 years (74.3%), older adults aged 50–65 years (60.4%), followed by children < 2 years old (34.5%). The mean duration of hospital stay was 7 days and there was no statistically significant difference between the different age groups (*P*-value = 0.788). In total, 70 (4.0%) patients required ICU admission for management of influenza-related complications. The highest rates of these admissions were at both age extremes; 13.2% of the elderly ≥65 years and 8.0% of children < 2 years with a significant *P*-value < 0.001 between the different age groups. However, there was no significant difference in the mean duration of ICU stay for all age groups (*P*-value = 0.533). Oxygen supplementation was required for 121 patients (7.5%) of which 35 cases ultimately required mechanical ventilation. There was a significant statistical difference among different age groups concerning oxygen supplementation and mechanical ventilation with the highest percentages for oxygen supplementation requirement among the elderly ≥65 years (28.3%) while the highest percentages for mechanical ventilation was among children < 2 years (4.7%).

Since follow up was not documented for outpatients, outcome was only reported in 469 hospitalized patients, out of which 18 cases (3.8%) died during their hospital stay. Influenza type A was the detected type in 15 out of the 18 mortality cases, while Influenza type B was detected in the remaining 3 cases. Eleven cases (69%) were older than 50 years and three cases were young adults. Only 4 cases were in the pediatric age group (< 5 years old) with influenza A detected in all four cases. All cases with fatal outcome had at least one comorbid condition except for one case who was one month old. This case was also co-infected with respiratory syncytial virus. Another 7 cases also had laboratory proven viral co-infections (Cytomegalovirus (CMV), Epstein Barr Virus (EBV) and Herpes Simplex Virus Type-1 (HSV-1); five of whom had an underlying malignancy, one had an underlying neurological disease and one was a healthy pregnant female.

Table 4 shows risk factors for increased mortality among hospitalized patients due to influenza infection. A statistically significant *P*-value was found for moderate to severe disease, radiologically-confirmed pneumonia, and presence of viral or bacterial co-infection. Other statistically significant risk factors included neurological disorder (0.002) and malignancy (0.043). There was no statistically significant difference in mortality among different age groups or types of influenza infection detected. However, when multivariable logistic regression was done, only the presence of a proven viral co-infection, bacterial co-infection and underlying neurological diseases were found to be independent risk factors for increased mortality (Table S2).

**Table 4:** Risk factors for mortality in cases of laboratory proven influenza in hospitalized patients

Characteristic	Recovery, n (%) *N* = 451	Death, n (%) *N* = 18	*p*-value
**Age group**			0.909*
*[0–2 years [*	54 (12.0)	2 (11.1)	
*[2–5 years [*	33 (7.3)	2 (11.1)	
*[5–10 years [*	24 (5.3)	0 (0.0)	
*[10–19 years [*	24 (5.3)	0 (0.0)	
*[19–50 years [*	99 (22.0)	3 (16.7)	
*[50–65 years [*	93 (20.6)	5 (27.8)	
*≥ 65 years*	124 (27.5)	6 (33.3)	
**Gender**			0.611
*Male*	228 (50.6)	8 (44.4)	
*Female*	223 (49.4)	10 (55.6)	
**Presence of underlying conditions**	318 (70.5)	16 (88.9)	0.091
**Underlying medical conditions**			
*Cardiovascular disease*	161 (35.7)	8 (44.4)	0.449
*Chronic pulmonary disease*	43 (9.5)	1 (5.6)	1.000*
*Asthma*	39 (8.6)	2 (11.1)	0.665*
*Immunosuppressive state (other than malignancy)*	15 (3.3)	1 (5.6)	0.471*
*Diabetes Mellitus*	75 (16.6)	3 (16.7)	1.000*
*Renal disease*	45 (10.0)	3 (16.7)	0.414*
*Neuromuscular disorders*	9 (2.0)	1 (5.6)	0.326*
*Neurological disorders*	34 (7.5)	6 (33.3)	0.002*
*Malignancy*	101 (22.4)	8 (44.4)	0.043*
*Hemoglobinopathy*	15 (3.3)	0 (0.0)	1.000*
**Type of influenza**			1.000*
*Influenza A*	363 (80.5)	15 (83.3)	
*Influenza B*	79 (17.5)	3 (16.7)	
*Co-infection A&B*	9 (2.0)	0 (0.0)	
**Moderate to Severe disease (*N*** **= 466)**	269 (60.0) ^†^	18 (100.0)	**0.001**
**Radiologically confirmed pneumonia (*N*** **= 418)**	173 (43.3)^a^	17 (94.4)	**< 0.001**
**Proven viral co-infection**^b^	13 (2.9)	7 (38.9)	< 0.001*
**Proven bacterial co-infection**^c^	94 (20.8)	11 (61.1)	0.001*

*Pearson’s Chi-Square test was used (no expected count less than 5)*

*Fisher’s exact test was used when expected count was less than 5

^†^3 patients in this group had missing data

^a^51 patients had no imaging results

^b^Proven viral co-infection was defined by a positive PCR for other viruses whether from respiratory or other specimens

^c^Proven bacterial co-infection was defined by the presence of a positive culture

### Antibiotic and antiviral use

Antibiotics were prescribed for 494 patients (41% of those with available data *N* = 1202) with laboratory-confirmed influenza infection. Only 102 (20.6%) of those who received antibiotics had a proven bacterial co-infection. A proven bacterial co-infection was considered in the presence of a positive culture. In fact, pneumonia cases accounted for 49% of antibiotic prescription though not all of these cases were proven to be bacterial.

Antivirals were prescribed for 821 patients (68.8% of those with available data *N* = 1193) with laboratory-confirmed influenza. Oseltamivir was prescribed in 807 subjects while zanamivir was prescribed in the remaining 14 patients. Table (S3) describes the different characteristics of the patients who received antiviral treatment. The majority of elderly patients and children below 2 years of age received antiviral therapy. There was a statistically significant higher percentage of antiviral prescription among inpatients, pregnant females and those with comorbid conditions.

### Influenza hospitalization rates

**Fig. 2:**
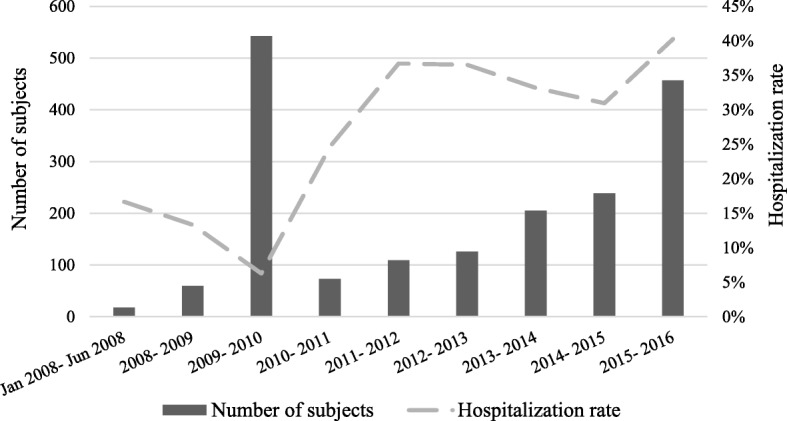
Influenza-associated hospitalization rate over the seasons

Overall influenza-associated hospitalization rate reached 26.6%. The annual influenza-associated hospitalization rate was lowest during the 2009 pandemic season (6.8%) and trended up gradually to reach the highest during 2016 (41%) (Fig. 2). In addition, the monthly influenza-associated hospitalization rate was as low as 3% in July and reached almost 37% during January.

## Discussion

Our results show that seasonal influenza causes a substantial burden in the Lebanese population. The AUBMC serves an annual average of around 400,000 persons including in-hospital admissions, clinic visits and ED visits. A total of 11,288 patients were tested for influenza virus during the study period that extended over 8 influenza seasons of which 1829 (16%) patients were positive for either influenza A, B or both. A total of 475 (26.6%) subjects were hospitalized. On average, hospitalization rate attributable to influenza accounted for 0.24% annually. However, this number is likely an underestimate of the true influenza-related hospitalizations rate because influenza testing is done at the request of the physician and not every patient was tested for influenza. Therefore, a population based prospective surveillance study is needed to better estimate the burden of Influenza-related hospitalizations. The average annual positivity rate (the number of positive tests divided by the total number of requested tests) for tested patients was 14% and this is similar to the positivity rate (13.4%) reported by the Lebanese SARI Sentinel Surveillance conducted by the Ministry of Public Health for the 2015/2016 season [37]. Moghoofei et al. estimated an influenza infection prevalence of 10% in the Middle East that approaches our positivity rate in Lebanon [38]. In his systematic review, 71 studies were included of which only one study was from Lebanon but included a very small sample size [30].

According to the WHO Global Epidemiological Surveillance Standards for Influenza, a standard age grouping into 6 groups is suggested for analysis [5]. We further subdivided the population into an additional 7th ‘adolescent’ age group (10–19 years). Results from the SARI surveillance by the MOH showed a similar distribution, with higher positivity rates among those 15–49 years reaching 26% in the southern sentinel site and a positivity rate of 21% for those 5–15 years in the Beirut sentinel site [37]. According to a cross-sectional review published in 2015, influenza vaccine uptake among Lebanese adults was only 27.6% during the 2014/2015 season [39]. The vaccination rate for our population reached 22.4%. Unfortunately, we did not have vaccination data for most of our paients and the majority of available data was for pediatric patients. It is important to recall that although influenza vaccines were thought to be mostly effective for children 2 years and older, recent data showed high vaccine efficacy reaching 60% in infants as young as 6 months [36, 40–44].

Our analysis showed that all severity parameters, except for hospital and ICU stay duration, were highly present within two age groups: below 2 years and ≥ 65 years. These results demonstrate that extremes of age have a more severe course of disease including a higher mortality rate. Several recommending bodies consider these age groups at higher risk for severe disease and mortality and thus recommend early initiation of antiviral therapy and emphasize the importance of vaccination [45, 46]. Unfortunately, vaccination is not recommended for these age groups nor included in the national immunization program in Lebanon [47].

The presence of at least one co-morbidity, mainly cardiovascular diseases, asthma, malignancy and diabetes mellitus, in 71% of hospitalized patients and 31% of outpatients is worrisome especially with recent data on influenza being a trigger for exacerbation of these illnesses and vice versa. Several papers have showed an association between influenza infection and myocardial infarction, chronic obstructive pulmonary disease exacerbations and diabetic crises [18, 23, 24, 27, 48, 49]. This also means that at least half of the study population were probably at higher risk for severe influenza disease and outcome including death [50].

Several complications have been linked to influenza infection including both pulmonary and extra-pulmonary. Both bacterial and viral pneumonias are established complications of influenza infection and have been reported as an independent risk factor for increased severity and mortality [51–53]. Our review showed that radiologically confirmed pneumonia was a risk factor for increased mortality among hospitalized patients (Table 4); however, it was not found to be an independant risk factor for mortality in the multivariable logistic regression (Table S2). It was the most common complication reported with highest incidence in older age groups > 50 years. We could not determine whether these were primary viral pneumonias or bacterial co-infection in most of the cases. Inflammatory markers including C-reactive protein and procalcitonin were not used regularly especially in outpatients, so we did not track them. Regardless, 89% of these cases received antimicrobial treatment according to the treating physician’s judgment. Of these pneumonia cases, 16 (6.6%) progressed to acute respiratory distress syndrome (ARDS).

Our population also displayed a wide array of extra-pulmonary complications including myocardial infarction, encephalopathy, shock, renal failure and liver failure. These complications have been reported in several articles [54–59] and interest in determining their real burden is increasing as these extra-pulmonary complications actually represent a significant burden related to influenza infection that is sometimes overlooked [55].

According to the global burden of diseases study, influenza accounted for 0.26% of all-cause mortality globally. It also estimated that 0.6 per 100,000 population died from influenza in Lebanon [3]. In our review, we identified 18 fatalities representing 3.8% of all hospitalized laboratory proven influenza cases during an 8-year period. In Egypt, according to influenza surveillance data from 2007 to 2014, influenza positivity was 17% among SARI cases with a mortality rate reaching 2% [60]. In Oman, influenza-associated deaths from 2012 to 2015 were estimated at 0.9 per 100,000 population and was highest among those ≥65 years (18.6 per 100,000 population) [61]. This was similarly reported by the Global Burden of Diseases study where the population attributable fraction (PAF) for adults older than 70 years was 6.3% compared to 2.9% for children younger than 5 years [3]. This is similar to our data where the highest mortality was in those ≥65 years (3.6%) compared to 1.5% in those below 2 years. According to our results, factors found to be associated with increased mortality were underlying neurological disease, malignancy, moderate to severe disease, proven viral or bacterial co-infection, and presence of pneumonia (*P*-value < 0.05). However, after multivariate logistic regression, only the presence of a proven viral or bacterial co-infection, pneumonia and neurological disease were independent risk factors for increased mortality. Unlike the data from Egypt [60], we didn’t find any association between influenza type and mortality. A systematic review by Klein et al. showed a very variable range in the incidence of bacterial co-infection with influenza from 2 to 65% in different studies [62]. It is however associated with an increased incidence of morbidity and mortality in all age groups for both seasonal and pandemic influenza with *Staphylococcus aureus* and *Streptococcus pneumoniae* being the most common pathogens identified [62, 63]. With the advance in the detection methods used to identify causative viral pathogens, several papers have discussed the increasing incidence of viral co-infection and its role in the severity of influenza infection [64, 65]. Though our results did show a higher rate of viral co-infection in the mortality cases, most of these viruses were not respiratory viruses. With the advent of routine use of a multiplex PCR respiratory panel at many centers around the world, more cases of co-infection with other respiratory viruses are likely to be detected.

According to the recent guidelines by the Infectious Diseases Society of America (IDSA), antiviral treatment should be started when influenza infection is suspected or confirmed in any hospitalized patient, those with severe or progressive illness, in children ≤2 years and adults ≥65 years old, pregnant females and 2 weeks postpartum, extremely obese patients, children and adolescents receiving salicylate-containing medications, patients with chronic medical conditions including immunosuppression and residents of chronic care centers [46]. Treatment should also be considered for contacts of high-risk patients mentioned above (household or healthcare providers) and when symptoms’ onset is ≤2 days at presentation. When treatment is warranted, a single neuraminidase inhibitor (NAI) should be used [46, 66]. Oseltamivir was almost exclusively used in our population. It was prescribed for almost 70% of the patients. Our review period, however, includes the 2009 pandemic when NAI’s were used extensively; but even when excluded in the analysis, the rate of antiviral use remains high (73%). Our data shows that there was a tendency to follow the guidelines though maybe not completely. Antiviral prescription was significantly higher at the extremes of age reaching 96% in ≥65 years, 95% of hospitalized patients, 84% of patients with underlying high-risk conditions and 84% of pregnant females. Nonetheless, it seems that the duration since onset of symptoms and the severity of disease per se did not influence the decision to initiate antivirals. A more detailed description of each case would further evaluate the level of adherence to the guidelines in order to implement a judicious plan for the use of these antivirals and try to control influenza resistance. On the other hand, prompt use of antivirals may reduce unnecessary use of antibiotics by decreasing the risk of bacterial co-infections, which are reported to be as low as 2% [46, 67, 68]. This implies the need for a more thorough evaluation before the decision to start antibiotics due to the increasing reports of resistant organisms mainly MRSA [62, 69]. Nonetheless, recommendations provided by the IDSA emphasize the use of antibiotics empirically in severe cases of influenza and in those who deteriorate after initial improvement [46]. Antibiotics were prescribed in around 40% of patients, half of which had an underlying pneumonia. A similar rate of antibiotic prescription was reported in Algeria (41%) [70] and Switzerland (45%) [71].

Both influenza A and B co-circulated during the 8 seasons with influenza A being predominant during each season. Historically, there has been more interest in researching influenza A virus mainly because of its capability to cause pandemics and underestimating the burden of influenza B virus. However, our data shows an increased morbidity associated with influenza B evident mainly by the higher incidence of ICU admissions although with no difference in mortality incidence for both types. Earlier published data have shown an increasing burden for influenza B in the USA, [72] whereas data from other countries showed a contradicting role of type B in influenza related morbidity and mortality [60, 72–75]. Further population-based studies are needed to look further into the real burden of Influenza B and comparing its clinical characteristics to influenza A.

In the MENA region, there is limited data on the circulation patterns of both influenza A and B. It is of paramount importance to determine the timing of influenza circulation for each region in order to decide on the best timing for vaccine delivery, aid healthcare workers in their clinical management of suspected cases in addition to increasing emphasis on prevention of infection and control of transmission [76]. In his review of surveillance data from the MENA region for the time period 2010–2016, Caini et al. reported that most of the region’s countries had a primary peak between January and March with no or barely noticeable secondary peaks [77]. According to the Lebanese SARI sentinel surveillance, the primary peak extended from January till mid of March [37]. This is consistent with our data, which shows that the influenza season starts by November–December and peaks during January and February. These results imply that vaccination should be administered between September and October of each year to ensure optimal protection.

Our study has several limitations. The most significant limitation is the fact that this data was collected retrospectively from chart review. We could not obtain accurate data on vaccination rates in addition to other missing or incomplete data. Another limitation is that all these cases reviewed were from a single center that, despite being a major tertiary medical center, cannot reflect the actual Lebanese burden of influenza. A third limitation is the fact that we included all laboratory proven influenza cases regardless of initial presentation symptoms or reason for admission. As a result, we could not calculate influenza-attributable SARI cases and we could not compare this to other published data. Also, we did not exclude possible health-care associated infections, although they contribute to the burden of influenza. Moreover, mortality rate associated with influenza might not be accurate since we had no follow up data on outpatients and thus significant data was missing.

## Conclusion

This study is a first attempt to estimate the burden of influenza in Lebanon including a large number of patients. Despite being a retrospective review, results offer groundwork for further influenza research. Based on the results presented by our review, influenza causes a substantial number of hospitalizations annually in Lebanon with significant morbidity and mortality across all age groups. Even though we had incomplete data on vaccination, a rate of 22% is fairly low. Fortunately, there is an increased interest in influenza research and surveillance along with increased testing for ILI and SARI cases. The SARI sentinel surveillance initiated by the Ministry of Public Health since 2015 promises to provide better data on the burden of influenza on a national level thus aid in developing a better vaccination program and maybe incorporate it into the national immunization program. A population based prospective surveillance study is needed to better estimate the burden of influenza in Lebanon which would help to drive policy on influenza control.

## Supplementary information

**Additional file 1: Table S1.** Presenting symptoms of laboratory-confirmed influenza cases according to different age groups. **Table S2.** Results of multivariable logistic regression for independent risk factors associated with increased mortality. **Table S3.** Characteristics of patients receiving antiviral therapy over the study period.

## Data Availability

The datasets used and/or analysed during the current study are available from the corresponding author on reasonable request.

## Abbreviations

WHO: World Health Organization
MENA: Middle East and North Africa
ILI: Influenza-like illness
SARI: Severe Acute Respiratory Infection
AUBMC: American University of Beirut Medical Center
CIDR: Center for Infectious Diseases Research
RDT: Rapid Diagnosis Test
PCR: Polymerase Chain Reaction
CRF: Case Report Form
CT: Computed Tomography
