# Supplementary material for: The burden of laboratory-confirmed influenza infection in Lebanon between 2008 and 2016: a single tertiary care center experience
Source: BMC Infect Dis. 2020 May 12;20:339. doi: 10.1186/s12879-020-05013-7 (PMC7216128; doi:10.1186/s12879-020-05013-7)
Supplement: Supplementary file 1 — Additional file 1: Table S1. Presenting symptoms of laboratory-confirmed influenza cases according to different age groups. Table S2. Results of multivariable logistic regression for independent risk factors associated with increased mortality. Table S3. Characteristics of patients receiving antiviral therapy over the study period. [file 12879_2020_5013_MOESM1_ESM.docx]

**Table S1.** Presenting symptoms of laboratory-confirmed influenza cases according to different age groups

|  | **Total** | **[0-2[** | **[2-5[** | **[5-10[** | **[10-19[** | **[19-50[** | **[50-65[** | **≥65** | **p-value** |
| --- | --- | --- | --- | --- | --- | --- | --- | --- | --- |
|  | **n (%)** | **n (%)** | **n (%)** | **n (%)** | **n (%)** | **n (%)** | **n (%)** | **n (%)** |  |
| **Fever (N=1408)** | 1262 (89.6) | 136 (88.9) | 188 (94.9) | 202 (95.3) | 199 (92.1) | 280 (88.9) | 125 (84.5) | 132 (79.5) | **<0.001** |
| **Generalized symptoms (N=1399)** | 683 (48.8) | 19 (12.0) | 50 (25.4) | 95 (45.5) | 125 (60.4) | 209 (66.6) | 94 (63.5) | 91 (54.8) | **<0.001** |
| **Respiratory symptoms (N=1401)** | 1347 (96.1) | 141 (91.6) | 189 (95.5) | 207 (96.7) | 199 (95.2) | 306 (98.1) | 146 (98.6) | 159 (95.8) | **0.015** |
| **Gastrointestinal symptoms (N=1383)** | 466 (33.7) | 52 (35.1) | 65 (33.7) | 79 (37.4) | 65 (31.4) | 120 (38.7) | 48 (32.4) | 37 (22.3) | **0.019** |
| **Others (N=1369)** | 186 (13.6) | 25 (17.1) | 20 (10.6) | 23 (11.1) | 17  (8.2) | 40 (13.0) | 29 (19.7) | 32 (19.3) | **0.005** |

Pearson's Chi-Square test was used (no expected count less than 5).

Generalized symptoms: Headaches, myalgia, malaise, arthralgia or dehydration; Respiratory symptoms: Cough, runny nose, sore throat, tachypnea, dyspnea, apnea, respiratory discomfort, or wheezing; Gastrointestinal symptoms: Diarrhea, nausea, vomiting, or abdominal pain; Others: Neurological symptoms, conjunctivitis, confusion, hallucination.

To note that a different denominator was used to calculate the rate of each variable. The denominator depended on the number of cases with available data on the studied variable.

**Table S2.** Results of multivariable logistic regression for independent risk factors associated with increased mortality

| **Risk factors** | **ORa [95% CI]** | **p-value** |
| --- | --- | --- |
| **Neurological disorders** | 5.2 [1.5-18.3] | **0.009** |
| **Proven bacterial co-infection** | 3.3 [1.1-9.9] | **0.037** |
| **Proven viral co-infection** | 8.0 [2.2-28.5] | **0.001** |

ORa: adjusted Odds Ratio; CI: Confidence Interval.

Included variables: Neurological disorders, malignancy, moderate to severe disease, radiologically confirmed pneumonia, proven bacterial co-infection, proven viral co-infection

**Table S3.** Characteristics of patients receiving antiviral therapy over the study period.

|  | **Antiviral use, n (%)** | **p-value** |
| --- | --- | --- |
| **Age groups** |  | **<0.001** |
| *[0-2 years[ (N=120)* | 97 (80.8) |  |
| *[2- 5 years[(N=150)* | 72 (48.0) |  |
| *[5-10 years[(N=161)* | 70 (43.5) |  |
| *[10-19 years[ (N=160)* | 79 (49.4) |  |
| *[19- 50 years[ (N=296)* | 219 (74) |  |
| *[50-65 years[ (N=144)* | 129 (89.6) |  |
| *≥ 65 years (N=162)* | 155 (95.7) |  |
| **Days since onset of symptoms** |  | 0.339 |
| *≤2 days (N=727)* | 508 (69.9) |  |
| *>2 days (N=295)* | 295 (67.2) |  |
| **Setting** |  |  |
| *Inpatients (N=467)* | 443 (94.9) | **<0.001** |
| *Outpatients (N=725)* | 378 (52.1) |  |
| **Comorbid conditions** |  | **<0.001** |
| *Yes (N=550)* | 463 (84.2) |  |
| *No (N=639)* | 355 (55.6) |  |
| **Moderate to severe disease** |  | 0.059 |
| *Yes (N=527)* | 377 (71.5) |  |
| *No (N=661)* | 439 (66.4) |  |
| **Pregnant** |  | **0.002** |
| *Yes (N=64)* | 54 (84.4) |  |
| *No (N=169)* | 108 (63.9) |  |

Pearson's Chi-Square test was used (no expected count less than 5).
